# High complication rate in the early experience of minimally invasive total hip arthroplasty by the direct anterior approach

**DOI:** 10.3109/17453674.2012.711701

**Published:** 2012-08-25

**Authors:** Anne J Spaans, Joost A A M van Hout, Stefan B T Bolder

**Affiliations:** Department of Orthopaedic Surgery, Amphia Hospital, Breda, the Netherlands

## Abstract

**Background and purpose:**

There is growing interest in minimally invasive surgery techniques in total hip arthroplasty (THA). In this study, we investigated the learning curve and the early complications of the direct anterior approach in hip replacement.

**Methods:**

In the period January through December 2010, THA was performed in 46 patients for primary osteoarthritis, using the direct anterior approach. These cases were compared to a matched cohort of 46 patients who were operated on with a conventional posterolateral approach. All patients were followed for at least 1 year.

**Results:**

Operating time was almost twice as long and mean blood loss was almost twice as much in the group with anterior approach. No learning effect was observed in this group regarding operating time or blood loss. Radiographic evaluation showed adequate placement of the implants in both groups. The early complication rate was higher in the anterior approach group. Mean time of hospital stay and functional outcome (with Harris hip score and Oxford hip score) were similar in both groups at the 1-year follow-up.

**Interpretation:**

The direct anterior approach is a difficult technique, but adequate hip placement was achieved radiographically. Early results showed no improvement in functional outcome compared to the posterolateral approach, but there was a higher early complication rate. We did not observe any learning effect after 46 patients.

In recent years, there has been growing interest in minimally invasive surgery (MIS) techniques for total hip arthroplasy (THA). A number of articles have described the operation using smaller incisions and different approaches. The advantages of these MIS techniques are considered to be reduced soft tissue trauma, reduced blood loss, less postoperative pain, shorter hospital stay, a better cosmetic appearance, and faster recovery time (Howell et al. 2004, Siddiqui et al. 2005, Sendtner et al. 2010, Bergin et al. 2011). However, some studies have also shown a worse outcome using these approaches (Pagnano et al. 2005, D’Arrigo et al. 2009, Woolson et al. 2009). Furthermore, tissue-sparing surgery may result in a higher complication rate, particularly in the so-called “learning curve” period—the first 20 cases for a single surgeon (D’Arrigo et al. 2009).

The soft-tissue-preserving nature of the direct anterior approach (DAA) combined with the relatively low risk of dislocation has generated interest in this approach for hip replacement over the last decade (Siguier et al. 2004, Rachbauer and Krismer 2008). Safe and reliable placement of the implants can be obtained, and muscle strength was found to be improved up to 2 years after THA using an anterior approach rather than an anterolateral approach (Mayr et al. 2009). Light and Keggi (1980) described the technique of using the anterior approach with a curved transverse skin incision. It was basically a modified Smith-Peterson approach to the hip joint (Light and Keggi 1980, Oinuma et al. 2007). This approach is muscle-splitting, because it uses the intermuscular plane between the gluteus medius and the tensor muscles laterally, and the sartorius and rectus muscles medially. Additionally, the DAA is the only true internerve approach between the zones of innervation of the superior and inferior gluteal nerves laterally and the femoral nerve medially. As a result, the risk of limping as with the anterior and lateral approaches can be reduced and the higher risk of dislocation associated with posterior approaches is avoided (Lowell and Aufranc 1968, Siguier et al. 2004, Lovell 2008, Bender et al. 2009).

Acetabular exposure is relatively easy, with excellent visualization. There is a very small danger of injuring the sciatic or femoral nerve. Disadvantages include the difficulty in doing straight reaming, the femoral exposure, and possible damage to the lateral femoral cutaneous nerve (Lowell and Aufranc 1968, Light and Keggi 1980, Siguier et al. 2004, Oinuma et al. 2007, Bender et al. 2009, Goulding et al. 2010).

Regarding early complications, the hypothesis to be tested was that patients operated for a THA through a DAA would have a more rapid early recovery and better postoperative results than patients operated through our regular posterolateral approach (PLA). We also examined our learning curve with this new technique.

## Patients and methods

In the period January through December 2010, 46 patients were operated with THA using the DAA. All patients were followed after surgery. Inclusion criteria were primary osteoarthritis in patients with a BMI of < 35, for adequate visibility with an MIS approach. Exclusion criteria were secondary osteoarthritis, tumors, hip deformities, and previous surgery to the affected hip. All the operations were performed by 2 surgeons (JH and SB), who operated together on all patients. Before starting this operation technique, the surgeons had an internal education and training on cadavers that was supervised by an experienced orthopedic surgeon who had used the DAA for 5 years. This surgeon also assisted at and supervised the first 4 operations performed by JH and SB in our own hospital. All DAA patients were included in the study, including the first 4 supervised cases.

The DAA patients were compared to a matched cohort of 46 patients who were treated with THA by a posterolateral approach (PLA) because of primary osteoarthritis. The patients were matched by age and co-morbidities. The distribution of sexes in the DAA group was almost equal, whereas there were more female patients in the PLA group. The BMI was statistically significant higher in the PLA group (Table 1).

**Table 1.** Demographic data

	DAA group n = 46	PLA group n = 46	p-value
Mean (SD) age intraoperatively	69 (9.8)	68 (11)	0.9
Sex			0.06
Male	24	14	
Female	22	32	
Mean BMI (SD)	25 (3.0)	29 (4.3)	< 0.001
Preoperative functional score			
Harris hip score	57	55	0.2
Oxford hip score	20	18	0.2
ASA classification			0.7
1	7	9	
2	35	29	
3	4	8	
4 / 5	0	0	

DAA: direct anterior approach. PLA: posterolateral approach.

### Operation technique

We operated the patients strictly according to the operation technique described by Rachbauer and Krismer (2008). Uncemented femoral and acetabular components were used in all patients. This included a Trident cup, an Accolade TMZF stem, a highly crosslinked polyethylene X3 liner, and a cobalt chrome 36-mm head (all Stryker Orthopaedics, Mahwah, NJ). The implant size used was chosen by the surgeon as the most appropriate for each patient after preoperative digital templating had been performed.

### Postoperative care

All patients received the same standardized postoperative rehabilitation protocol, which included antibiotics for 24 h, antithrombotic prophylaxis for 6 weeks postoperatively, and physiotherapy from the first day after surgery with full weight bearing. Patients were either discharged home or transferred to a rehabilitation facility based on their medical condition, progress in therapy, and home support system.

### Evaluation

We evaluated the short-term results in the DAA and PLA groups. Operative, clinical, and radiographic outcomes and early complications were analyzed.

In the DAA group, we evaluated the learning effect of the operative outcome by dividing the group into 3 subgroups. The first hip placement with DAA at our center was included. The 3 subgroups were: hips 1–15 (subgroup 1), hips 16–30 (subgroup 2), and hips 31–46 (subgroup 3). We compared operation time (skin incision to skin closure), intraoperative blood loss, time of hospital stay, clinical outcome, and complications in the 3 subgroups.

Clinical outcome was measured with the Harris hip score and the Oxford hip score questionnaires. All patients were scored preoperatively and at 6 weeks, 3 months, 6 months, and 1 year postoperatively. Radiographic analysis was done with standard anteroposterior pelvic and lateral views directly after surgery and at 6 weeks and 12 months, or if indicated for complications. These radiographs were evaluated independently by the same 2 senior orthopedic surgeons (JH and SB) for component position measured by leg-length difference, cup inclination angle, and stem alignment. Determination of leg length was done by measuring the vertical height from the teardrop line (a horizontal line drawn along the lower edge of the right and left acetabular teardrops, assuming pelvic symmetry) to a point chosen on the lesser trochanter. The vertical height to the same landmark was measured on the contralateral hip, and the difference was considered to be the postoperative leg-length discrepancy, with a maximum of 10 mm (Matta et al. 2005). Cup inclination was assessed by goniometric measurement of the angle between the teardrop line and the major diameter of the ellipse represented by the rim of the acetabular cup, with a target range of 35° to 55° (Matta et al. 2005). Stem position was evaluated by goniometric measurement of the angle subtended by the femoral shaft axis and the long axis of the femoral component on AP radiographs. Femoral component angulation between 3° varus and 3° valgus relative to the femoral shaft axis was considered “well-aligned” (Lewinnek et al. 1978, Dorr and Wan 1998, Sendtner et al. 2010).

### Statistics

Baseline information and information collected at each study visit was compared between each intervention group using the Student t-test. All p-values were 2-tailed, and the significance level was set at 0.05. The analyses were performed using SPSS software version 18.0.

## Results

### Operation time

The mean duration of the procedure was 84 min in the DAA group and 46 min in the PLA group (p < 0.001) (Table 2). The mean surgical time decreased with increasing experience, although not statistically significantly (Table 3).

**Table 2.** Operative outcome. Numbers are mean (SD)

	DAA group	PLA group	p-value
Blood loss, mL	704 (426)	364 (174)	< 0.001
Operation time, min	84 (28)	46 (9)	< 0.001
Hospital stay, days	4.8 (2.0)	4.7 (2.1)	0.8

### Blood loss

Mean intraoperative blood loss was 704 mL in the DAA group and 364 mL in the PLA group (p < 0.001) (Table 2). There was no reduction in the learning curve (Table 3). There were 7 patients in the PLA group and 27 patients in the DAA group who had blood loss of more than 500 mL.

**Table 3.** Effect of the learning curve in the DAA group. Numbers are mean

	Subroup 1	Subgroup 2	Subroup 3
Blood loss, mL	703	793	622
Operation time, min	96	80	77
Hospital stay, days	4.5	4.5	5.0

### Hospitalization

The mean length of hospital stay was 4.8 days in the DAA group and 4.7 days in the PLA group, without any reduction in the learning curve (Tables 2 and 3).

### Clinical outcome

Harris hip scores and Oxford hip scores were similar between the groups. In both groups, there was a statistically significant difference between pre- and postoperative measurements. There was no statistically significant difference in clinical outcome between the DAA group and the PLA group (data not shown).

### Complications

No superficial or deep wound infections and no deep venous thrombosis or nerve palsies (including the sciatic, femoral, or lateral cutaneous nerve of the thigh) occurred. The overall rate of other complications was higher in the DAA group (Table 4).

**Table 4.** Complications

	DAA group	PLA group
Intraoperative complications with conversion		
to the posterolateral approach		
Insufficient visibility	1	
Insertion of a cemented prothesis	1	
Acetabular fissure	1	1
Trochanteric fracture	1	
Postoperative complications		
Periprosthetic fracture		1
Dislocation	1	1
Revision		
cup migration / luxation	2	
femoral stem collapse	1	
M. quadriceps weakness	1	
Other complications		
Ischemic cerebrovascular accident	1	
Pneumonia	1	
Ogilvie syndrome		1

In the DAA group, 4 patients required an intraoperative conversion from a DAA to a PLA. After exposure of the hip using a DAA, the anterior wound was closed before THA was completed and the operation had to be finished by performing a PLA. The results of these 4 patients were included in the DAA group. In 2 of these patients there was technical error—inadequate visibility in one case and the need for cementing in the other. In 2 cases, the reason for conversion was a fracture and the need for a more extensile approach: in 1 case there was an acetabular fissure with the need for grafting and a cemented cup, and in 1 case there was a trochanteric fracture in femoral preparation with the need for placement of a hook plate. 1 patient had continuous weakness in the quadriceps muscle, even after more than a year of follow-up. We saw 2 early failures with the need for revision within 1 year for cup loosening and 1 early revision for femoral failure.

In the PLA group also, 1 patient had an intraoperative acetabular fissure that required a restrictive postoperative regime without acetabular grafting. 1 patient had a postoperative periprosthetic fracture within a year, after trauma.

1 patient in each group had a postoperative dislocation. In the DAA group, this happened in a patient who also had had an intraoperative conversion to a PLA.

### Radiographic evaluation

The prostheses in both groups had adequate placement without significant differences in cup inclination, leg-length difference, or femoral stem alignment (Table 5).

**Table 5.** Radiographic outcomes. Numbers are mean (SD)

	DAA group	PLA group
Cup inclination angle, degrees	49 (8.0)	46 (5.8)
Leg-length difference, mm	–0.64 (3.0)	0.51 (3.2)
Stem alignment, degrees	1.1 (1.2) varus	2.0 (1.4) varus

## Discussion

The strengths of our study include the consecutive series of patients, who were all subjected to the same preoperative and postoperative care protocols (regarding rehabilitation and pain). We included all patients operated with a DAA, including the very first one. We found that there was twice as much intraoperative blood loss, an almost doubled operation time, and more complications in the DAA group without any improvement in hospital stay or early functional outcome. There was only a decreasing trend in the learning curve associated with the operating time, but there was no effect regarding the amount of blood loss intraoperatively or the length of hospital stay, even after 46 cases.

There are many different approaches for THA, all with advantages and disadvantages. According to the recent literature, MIS techniques could—at least in theory—improve the results of conventional THA approaches, especially in the early postoperative period with reduced blood loss, tissue disruption, and hospital stay; they could give similar clinical outcomes and complication rates to those of conventional THA (Matta et al. 2005, Oinuma et al. 2007, Cheng et al. 2009, Mayr et al. 2009, Sendtner et al. 2010, Alecci et al. 2011).

The main advantage of the DAA would be that it follows a well-delineated intermuscular and internervous plane. It leaves the hip abductors and the posterior soft tissue envelope intact, potentially reducing the instability and dislocations associated with the release of these structures that is required in the posterior approach (Lowell and Aufranc 1968, Siguier et al. 2004, Lovell 2008, Bender et al. 2009). Also, the insertions of the gluteus minimus and gluteus medius remain intact, to reduce the postoperative limb and abductor dysfunction reported with the lateral and anterolateral approaches (Siguier et al. 2004, Rachbauer and Krismer 2008).

Good results have been achieved in some studies with the DAA for THA (Matta et al. 2005, Oinuma et al. 2007, Alecci et al. 2011). They found similar operating times to those in the present study (75–89 min), but less blood loss (350–393 mL) and fewer complications (in only 1.8–3.4% of their patients). Unfortunately, not all authors described (the effects of) their learning curve. Also, it is unclear whether their first patients were included in these studies.

Other studies have reported worse results of DAA (D’Arrigo et al. 2009, Woolson et al. 2009). They found a longer operating time (121–168 min) and more blood loss (858–1,344 mL). A high rate of complications (13–25%) was reported in their DAA groups. In the study by Woolson et al., the number of complications appeared to decrease after 30–50 operations. Also, we found a longer operation time in the DAA group, more blood loss, and no difference in length of hospital stay. Additionally, there were more complications in the DAA group.

Intraoperative complications and component misalignment could be more prevalent in MIS- THA due to the constrained surgical field, which makes visualization of anatomical landmarks and alignment of the components more challenging (D’Arrigo et al. 2009, Woolson et al. 2009). Intraoperative fracture of the proximal femur is the most frequent complication associated with minimally invasive techniques (D’Arrigo et al. 2009). We had 1 trochanteric fracture in the DAA group. We found an adequate positioning of the prosthesis with no differences between the PLA and DAA groups by measurement on conventional radiographs. Every new operation technique is associated with effort and often with a (temporary) increase in adverse events, the so-called learning curve (D’Arrigo et al. 2009, Krismer 2009). The learning curve is commonly thought to be on 20 cases for a single surgeon, but from our results, we believe that the learning curve for the DAA is at least more than 46 cases.

Based on our experience, we recommend that hip surgeons should be very careful in changing their daily routine and performing THA through a technique whose benefit has not been proven in the long term and which could cause an increase in complications, especially during the learning-curve phase. After 46 patients, we still did not see any evidence of further improvement with the DAA for THA. The complication rate after DAA may therefore be unacceptably high for some surgeons who already have a low complication rate with the conventional approach for hip replacement.
